# Teaming up to return to play: A shared responsibility to optimize care

**DOI:** 10.1016/j.hroo.2022.02.012

**Published:** 2022-02-22

**Authors:** Neel P. Chokshi, Rajat Deo

**Affiliations:** ∗ Sports Cardiology & Fitness Program, Division of Cardiovascular Medicine, Perelman School of Medicine at the University of Pennsylvania, Philadelphia, Pennsylvania; † Electrophysiology Section, Division of Cardiovascular Medicine, Perelman School of Medicine at the University of Pennsylvania, Philadelphia, Pennsylvania

In sports, officiating has become increasingly sophisticated and precise. Technological innovations have allowed the routine use of high-speed, 360-perspective video cameras to provide reliable verification of on-field calls. Games are often suspended so that referees can review film to ensure that the truth is maintained (when feasible) and judicious decisions are made. Although this approach takes considerably more resources, time, and coordination, it is a clear progression from the sometimes unilateral or paternalistic refereeing approach of the past, which often led to frustration and anger (think John McEnroe arguing a line call in tennis). As such, high-resolution imaging and video feedback often lead to a better experience for players, officials, and spectators. This process has also created more transparency around interpretation of the rules and, in turn, improved the integrity of the game. Similar opportunities exist to improve the process of evaluating student athletes with arrhythmic conditions in a systematic fashion to ensure greater transparency, communication, and understanding.

The process of screening, evaluation, and decision-making in cardiovascular risk assessment and return to play (RTP) in athletes with inherited arrhythmic disease is complex. Clinical evaluation requires access to expertise and testing that is geared toward the novel cardiovascular issues of the athlete. The care process itself often must be coordinated across different organizations or institutions, which are often geographically dispersed and can make communication challenging and incomplete. This specialized care must also take place in a timely manner that is distinct from traditional healthcare delivery. In short, the process is pressure-packed. Additionally, there are many stakeholders involved, including athletes, their families, cardiologists, team physicians, trainers, athletic administrators, and coaching staff. Each party has its own unique motives and concerns in R decisions. Further, a paucity of data and experience in these clinical scenarios leads to uncertainty and varying interpretations about the degree of risk.

In this issue, Shapero and colleagues^1^ examine the experiences of athletes and their families in navigating the process of RTP decision-making. They performed an in-depth qualitative analysis in a small group of athletes with predominantly inherited arrhythmic diseases or hypertrophic cardiomyopathy. Slightly more than half of this student-athlete cohort presented with cardiac arrest or syncope; and only a minority were identified through screening. Using a grounded theory approach, several themes emerged: the student athletes and their families found clinicians (1) to be paternalistic and unilateral in decision-making; (2) to lack clinical expertise in the cardiovascular condition and the implications of RTP; (3) to communicate poorly and lack understanding of the emotional toll of undergoing an evaluation; and (4) to express concern for personal liability. All subjects in this cohort were able to RTP in their respective sports, although some had to change institutions.

The findings highlight the challenges that athletes and families confront with either a serious cardiovascular diagnosis or abnormality identified through screening for a competitive sport. Many of these issues arise from an inexact understanding of the risk-benefit equation in the athlete’s eligibility. Assessing the risk calculus is complex for several reasons. First, risk is not binary but rather is a spectrum that is often based on symptoms, genetic results, and clinical phenotyping. Conveying risk as a spectrum to athletic institutions and patients can be ambiguous. Further, there are limited data on the exact risks for many of these cardiovascular conditions in the context of sports. This can often lead to differing opinions among clinicians and institutions on the suitability for RTP. In the absence of evidence, there has been a historically conservative approach to holding out athletes with disease. Fortunately, the body of literature continues to grow, with more studies and opinions suggesting that exercise can be safe in select scenarios.^2^ Further, in considering benefits, including emotional and physical, the importance of sports to these athletes is likely undervalued by many not routinely engaged with these clinical decisions.

To offset these challenges, we believe it is critical to develop strong working relationships between clinical specialists, team physicians, administrators, and, of course, student athletes and their families. This team-based, organized approach to risk assessment, monitoring, and communication was tested recently at our institution during the COVID pandemic. Early data suggested that individuals who had COVID may be at increased risk for developing myocarditis and arrhythmic complications.^3^^,^^4^ As students were returning to campus for athletic training, our arrhythmia and sports cardiology groups recommended specialized cardiovascular risk assessment in appropriate athletes. Prior to the resumption of formal team activities, we constantly reviewed the evidence for cardiac screening and our clinical experience linking cardiovascular manifestations of the post-COVID state. We simultaneously met with team physicians, administration, and athletic trainers to develop testing protocols that were widely disseminated to student athletes and their families prior to ongoing sports participation. In brief, our goal was to enhance communication and minimize the “surprise” factor to all, but especially to students and families, for the possibility of additional cardiac imaging and arrhythmia monitoring prior to RTP. Parents and students had an expectation of how the athletic season would evolve amidst the global pandemic prior to stepping foot in team facilities. We believe this approach created a unique level of transparency and built a prophylactic trust that could aid with communication, especially in complex cases.

Such lessons can help in designing a more patient-centric approach to the care of athletes with inherited arrhythmic disease. We need to leverage our improvements in arrhythmic risk stratification, monitoring, and genetic testing with colleagues who have expertise in the emerging field of sports cardiology. In addition to the clinical infrastructure that forms the basis for specialized care, it is equally important to partner with athletic departments and training staff to ensure that standard protocols for risk assessment are both uniform and timely across teams and athletes. Such a process will optimize transparency, equity, and trust between all stakeholders, especially our patients and their families.
